# Dynorphin B induces lateral asymmetric changes in feline spinal cord reflexes

**DOI:** 10.3389/fnins.2013.00244

**Published:** 2013-12-17

**Authors:** Alexander I. Pilyavskii, Waldemar Moska, Kazimir Kochanowicz, Natalia V. Bulgakova, Andriy V. Maznychenko, Inna V. Vereshchaka, Alexander I. Kostyukov

**Affiliations:** ^1^Department of Movement Physiology, Bogomoletz Institute of Physiology, National Academy of SciencesKiev, Ukraine; ^2^University of Physical Education and SportGdansk, Poland

**Keywords:** dynorphin B, naloxone, κ-opioid receptors, field potentials, spinal cord, segmental reflexes

## Abstract

The effects of dynorphin B (an agonist of κ-opioid receptors) and naloxone (an antagonist of opioid receptors) on the field potentials (FPs) evoked in the lumbar spinal cord of spinalized cats were examined following successive stimulation of pairs of identical peripheral nerves on both sides of the body. The FPs were recorded bilaterally using microelectrodes from symmetrical sites of the gray matter between the L6 and L7 segments of the spinal cord transected at level of Th11. Significant changes (up to 75%) were registered in the areas of the initial positive components of the FPs evoked by sequential stimulation of the *nn. gastrocnemius-soleus*, *flexor digitorum longus*, and *tibialis* at both hind limbs; a difference between the effects of various nerves was not observed. Two-Way ANOVA analysis showed that two factors, the injection type and recording side, as well as a combination of these factors, strongly influenced the amplitudes of the FPs. Statistically significant side- and injection-dependent differences were registered in the majority of the tests. Both the directions of the changes in the FPs and their relative amplitudes were not strongly connected with a definite side of the spinal cord in different animals. Therefore, it is possible to postulate that the κ-opioid receptors are distributed inhomogeneously over the neuronal populations transmitting the peripheral afferent signals from different hind limbs, thus indicating a possible presence of the lateral asymmetry effects.

## Introduction

In the spinal cord, the endogenous opioid system (EOS) may play a role in the transmission of sensory information, the regulation of motor reflexes and the potent modulation of the rhythmic pattern generator (Kondo et al., 2005; Blivis et al., 2007; Steffens and Schomburg, 2011). This system includes the three widely accepted opioid receptor types (μ, σ, and κ), which are referred and preferentially activated by beta-endorphin, enkephalins, and dynorphins, respectively. In the spinal cord, opioid peptides and receptors are expressed in the superficial layers of the dorsal horn and colocalize with sensory neurons. These entities are also expressed in the ventromedial areas of the gray matter (Weber and Barchas, 1983; Blivis et al., 2007; Schramm and Honda, 2010; Sardella et al., 2011). The μ-opioids have been found to decrease the amplitude of motor evoked potentials, the latency of the potentials increases through the inhibition of excitatory interneuronal pathways to the spinal motoneurons (Honda et al., 2012). In contrast, the opioid antagonists enhance spinal reflexes and control sensory inputs to spinal motor circuits (Clarke et al., 1998, 2001).

Analysis of the feline motor system has revealed a complex set of actions for opioids on transmission in the spinal motor reflex pathways. The μ- and σ- opioids have been shown to depress monosynaptic reflexes in the posterior biceps semitendinosus muscle by acting on the transmission of non-nociceptive effects from the flexor reflex afferents (FRA) to its motoneurons (Schomburg et al., 2011). In contrast, the specific excitatory nociceptive non-FRA pathways were mainly facilitated by these substances, which act predominantly on interneurons rather than on motoneurons. Presumably, the role of EOS in spinal motor control exceeds the mere suppression of nociceptive motor withdrawal reactions (Steffens and Schomburg, 2011).

The search for endogenous substances involved in “spinal memory” led to the identification of enkephalins and dynorphins as possible inducers of postural asymmetry. Neuronal networks regulated by opioid peptides showing side-specific actions may be part of the system regulating side-specific plastic responses to unilateral injury (Duggan and North, 1983). The phenomenon of postural asymmetry induced by endogenous opioid peptides has also been described in terms of skeletal muscle reactions (Chamberlain et al., 1963a,b; Bakalkin, 1989; Bakalkin and Kobylyansky, 1989), however, the underlying neurophysiological mechanisms of this phenomenon have not yet been identified.

The central effects of peptides with lateralized actions on transmission of the motor signal via ipsi- and contralateral interneurons and motoneurons in the spinal cord have not been studied in detail. In the present study, we performed a comparative analysis of the action of dynorphin B and naloxone on the ipsi- and contralateral intraspinal FPs induced by stimulation of the nerves of the left and right hind limbs in cats. Specifically, we would like to elucidate whether activation patterns of κ-opioid receptors with lateralized activities induce asymmetric motor response in the feline spinal cord with the goal of using this model for further neurophysiological analyses.

## Materials and methods

### Experiment preparation

The experiments were performed using nine adult cats (8 males, 1 female) weighing between 3.2 and 4.5 kg. The animals were purchased from a state-controlled animal farm through the common animal facility of A.A. Bogomoletz Institute of Physiology (Kiev). The use of the animals was approved by the Ethics Committee of the Institute and performed in accordance with the European Communities Council Directive of 24 November 1986 (86/609/EEC). The animals were initially anaesthetized with ketamine (Sigma), 25 mg/kg, intramuscularly. After insertion of a catheter into the external jugular vein, intravenous injections of pentobarbital sodium (40 mg/kg, Sigma) were used for anesthesia. Depending on the state of the animal, 1 ml of a 10 mg/ml solution was administered intravenously as necessary. The blood pressure was monitored with a catheter inserted into the common carotid artery. Laminectomy was performed over the lumbar enlargement of the spinal cord with an additional local opening over the lowest thoracic segment to follow the transection of the spinal cord. All hind limb nerves were cut in both of the hind limbs. The *nn. gastrocnemius-soleus* (GS), *flexor digitorum longus* (FDL), and *tibialis* (TIB) were also prepared for stimulation. The animal was suspended within a firm frame and the tibia bones of both hind limbs were fixed. Pools were formed above the exposed spinal cord from the skin flaps surrounding the hind limbs. The pools were filled with mineral oil and maintained at a temperature close to 37.5°C by means of radiant heat. The prepared nerves were placed over platinum hook electrodes for stimulation. The rectal temperature was maintained at a constant physiological level by heating the body with a controlled heating pad. When necessary, the cats were artificially ventilated to keep the end-tidal CO2 concentration at 3.8–4.5%. At the end of all of the experiments, the animals were sacrificed using an overdose of pentobarbital sodium (5 ml of a 60 mg/ml solution).

### Recording, data acquisition and analysis

Field potentials were recorded bilaterally by glass microelectrodes filled with 0.6 M K_2_SO_4_ that had a resistance of 0.6–0.8 MΩ. The input signals were amplified by two inputs from an Axoclamp 2A amplifier (Axon Instruments, USA). The microelectrodes were inserted into the spinal cord symmetrically with medially directed inclinations to vertical near 30°; the insertion points were located between the L7 and L6 dorsal roots, being shifted at 0.7–1.0 mm laterally from the dorsal root entrance. Deepness of the microelectrode insertions consisted 3.0–3.5 mm, what corresponded to the intermediate zone of the gray matter near the most dorsally located motoneuronal nuclei.

Output signals from the Axoclamp 2A were directed to two channels of a four-channel BrownLee 440 amplifier (BrownLee Precision, CA, USA); another set of two channels was used for recording the DSPs. The recorded signals (FPs, DSPs) were filtered within a 0.2–5000 Hz bandwidth, and stimulation marks were collected by a CED Power 1401 (Cambridge Electronic Design, UK). Spike 2 software (Cambridge Electronic Design, UK) was used for data acquisition and processing, and the input signals were digitized with a 12-bit resolution at the sampling rate of 10 kHz. The analyses were performed using Origin 8.0 software (OriginLab Corporation, USA). Four output channels of the CED Power 1401 interface were used to generate trigger pulses for six standard isolation units sending stimuli to the nerves. The isolation units were operated with a regimen of current control; the duration of stimuli was 0.2 ms. The stimulation intensities were defined in multiples of the threshold current for evoking the correspondent dorsal surface potentials and were adjusted to a range of 6–7 thresholds, which allowed activation of both the low- and high-threshold peripheral afferents. The sequences of the stimuli for the three pairs of nerves were as follows: *n*_1_(*l*); *n*_1_(*r*); *n*_2_(*l*); *n*_2_(*r*); *n*_3_(*l*); *n*_3_(*r*); where *n*_i_(*l*) and *n*_i_(*r*) (*i* = 1… 3) signify the identical nerves in the left and right hind limbs. The intervals between consecutive stimuli in the series were 0.8 s; the series of pulses were repeated ten times with 30 s intervals. Pairs of GS and FDL nerves were used to evoke FPs in six experiments. In three of the experiments examining these nerves, the FPs evoked by stimulation of the TIB nerve were also studied.

### Chemical stimulations

The methods used to deliver the active substances were as follows. For each animal, 500 μg of dynorphin B dissolved in 50 μl saline was delivered into the spinal cord with a microsyringe. The needle of the syringe was inserted 3 mm into the spinal cord vertically. The site of the injection coincided with the central sulcus and was 15 mm rostral to the recording microelectrodes. The needle tip was placed close to the central canal of the spinal cord. This method of substance delivery was quite effective and evoked a 3–5 min change in the FPs recorded more caudally. Naloxone (at 10 mg/kg) was dissolved in 2 ml saline and delivered by intravenous injection over a period of approximately one hour following the administration of dynorphin B. Naloxone was used instead of the known selective antagonists of κ-opioid receptors (Metcalf and Coop, 2005) that are predominantly slowly absorbed and eliminated, thus showing delay of the maximal effects by hours or days, compared to minutes for naloxone. In addition, naloxone also blocks μ- and δ-opioid receptors that have much higher affinity for this antagonist compared to κ-receptors. The used doses of naloxone exceed those that are ordinarily applied to block μ-opioids (Steffens and Schomburg, 2011).

## Results

**Figure 1:**
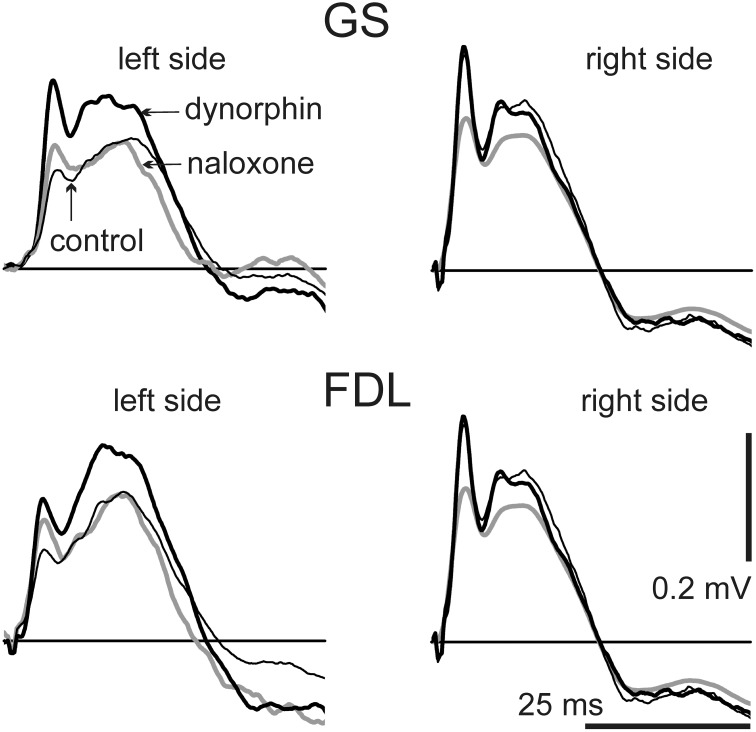
**Dynamics of changes in the field potentials (FPs) recorded bilaterally during consecutive stimulation of GS and FDL nerves in both hind limbs**. The averaged FPs (*n* = 10) are shown: before injections (*control*, thin black lines); within an interval 10–15 min after dynorphin B injection (*dynorphin*, thick black lines), and within an interval 10–15 min after naloxone injection (*naloxone*, gray lines). The single FPs serving for the averaging procedure were also used for quantitative assessment of the changes (Figures 2, 3; Tables 1, 2). For this purpose, there were defined areas of the initial positive components of single FPs (upward deflections of the signals lying above the straight lines of zero potential). Further quantitative treatments of the data for this experiment are presented under experiment N8 in Figures 2, 3 and in Tables 1, 2.

The present study analyses the FPs in sites located close to the lumbar motoneuronal nuclei and elongated in a rostro-caudal direction. Stimulation of the peripheral nerves activates homonymous motoneurons and numerous groups of interneurons via mono- and polysynaptic pathways. Microelectrodes can record complex potential oscillations and present summations of the evoked neuronal activation. Both the amplitude and form of the FPs varied on different sides of the spinal cord. Separate components of these potentials changed in different manners. The durations of the initial positive components in the FPs ranged over the 20–30 ms interval with the filtering parameters that were used (see Methods); for separate animals these durations were not changed noticeably throughout the experiment. These studies compared the average FPs (*n* = 10) recorded before dynorphin B injection (control) with similarly handled FPs recorded 10–15 min after dynorphin B injection as well as the FPs recorded within intervals 10–15 min after naloxone injection with FPs that were produced within intervals 60 min after dynorphin B injection. In the experiment presented in Figure 1, the dynorphin B and naloxone injections evoked different patterns of changes in FPs when recording on different sides of the spinal cord. On the left side, dynorphin B led to a marked rise in the amplitude of the FPs, whereas these potentials were almost unchanged on the right side of the spinal cord. Injection of naloxone diminished the test reactions almost symmetrically on both sides of the spinal cord. The characteristics of the FP changes after the two types of injections was quite similar for the GS and FDL stimulations; the basic features of these changes were not altered among the three stimuli (compare the left and right halves of Figure 1).

**Figure 2:**
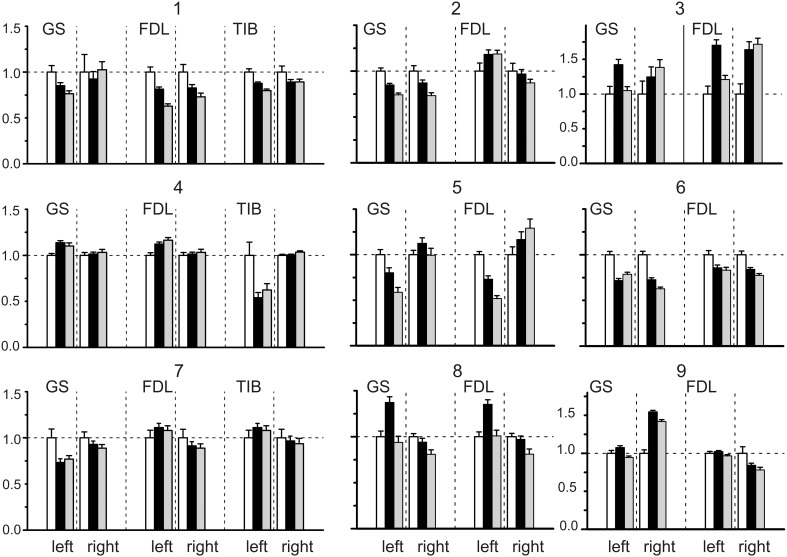
**Statistical parameters of the field potentials (FPs) recorded on both sides (left and right) of the spinal cord before (white bars) and after dynorphin B (black bars) and naloxone (gray bars) injections**. The normalized parameters of the three sets of FPs recorded bilaterally were compared within each experiment. Each set was connected with ten repetitions of the standard sequences of stimuli applied to the nerves of the left and right hind limbs in the control and after injection. Means ± SD of areas of the initial positive components of FPs (see Figure 1) were initially defined, then the obtained statistical parameters were normalized in respect to the mean value of the control set of the records on the given side. The following normalized statistical parameters are presented at Y axes of the graphs: 1 ± (SD_Con_ /*m*_Con_); (*m*_Dyn_ /*m*_Con_) ± (SD_Con_ /*m*_Con_); (*m*_Nal_ /*m*_Con_) ± (SD_Nal_ /*m*_Con_).

Changes in the amplitude of the FPs connected with the dynorphin B and naloxone injections sometimes were associated with a change in the duration of the initial positive components. In particular, this can be observed in the left panels of Figure 1, which presents the responses to stimulation of both the GS and FDL nerves on the left side. The temporal locations of extremes in the FPs could also change, and in many cases, the amplitude parameters of the FPs did not reflect their actual changes. For a comparison of the actions of the chemical stimulations, we used the areas under the first positive waves in FPs that lasted from the beginning until the transition into negativity. The results were similar to the average FPs shown in Figure 1; the positive components were also extracted from the single FP records, and their areas were used for statistical analysis of the changes evoked by the two types of injections (Figure 2, Table 1).

**Table 1:**
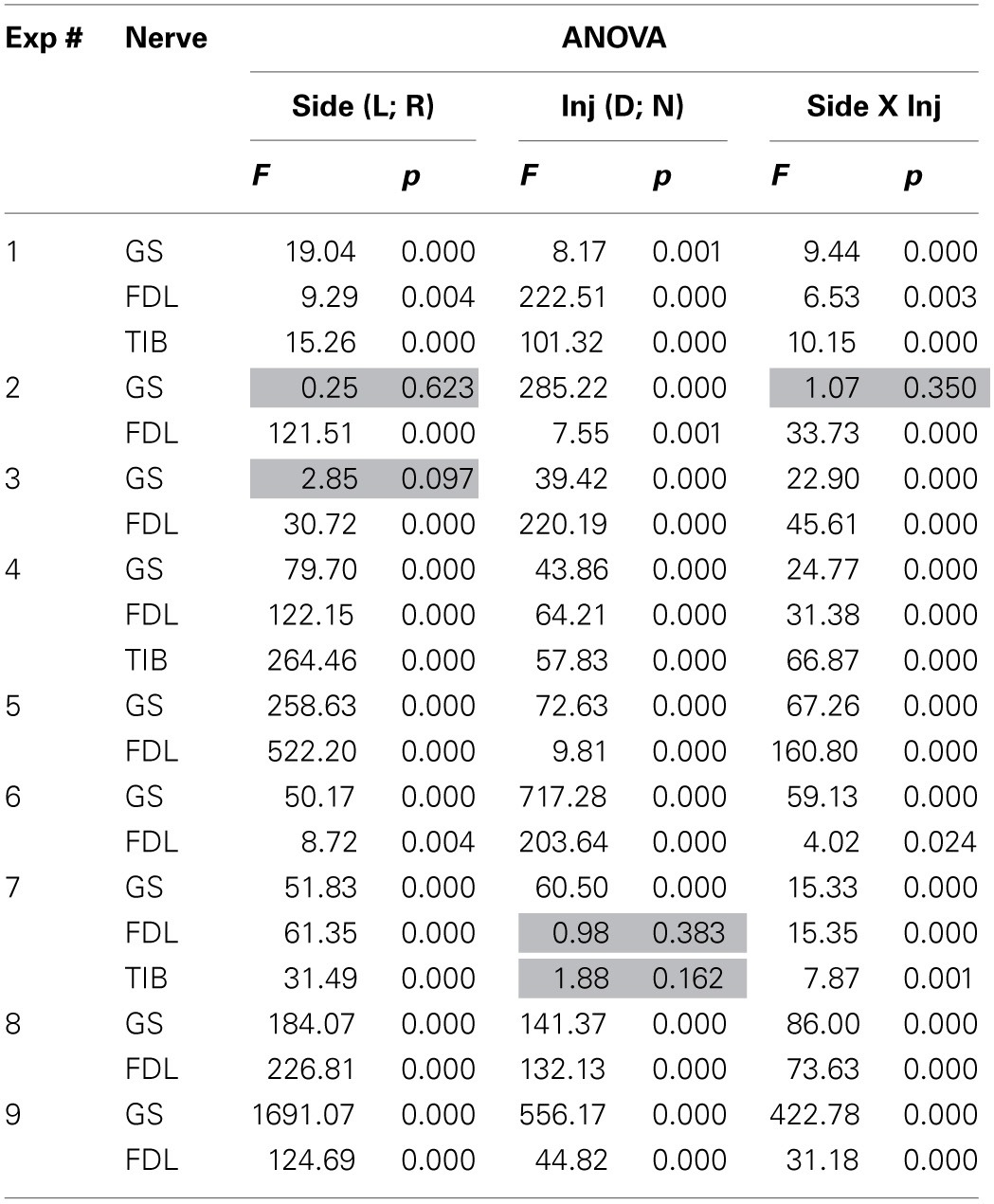
**ANOVA analysis of the data obtained in all of the experiments**.

*Two-way ANOVA analysis of the field potentials (FPs) recorded symmetrically in the left (L) and right (R)sides of the lumbar spinal cord during stimulation of the nerves of the corresponding hind limb. Compared are the normalized areas of the initial positive components of the FPs during 10 time repetitions of the identical stimulation patterns applied before injections (control tests); within 10–15 min interval after dynorphin B injection (D); or within 10–15 min interval after naloxone injection (N). The real dynamics of the changes in the parameters under study and explanation of the data normalization are presented in Figure 2. The dependence of FPs on the three following factors was analyzed: the side of recording (Side), the type of injection (Inj), and their interaction (Side X Inj). Absence of statistical significance at 0.05 level is marked by gray color. The same enumeration of experiments is shown in 2*.

The results of the statistical analysis of the changes evoked in the FPs by the dynorphin B and naloxone injections are presented in Figure 2. Due to a diversity of the amplitude and temporal parameters of the FPs recorded in various experiments, the following normalization procedure was applied for purposes of quantitative analysis. In each experiment, the normalized parameters for the three sets of FPs recorded bilaterally were compared. Each set consisted of ten repetitions of the standard sequences of stimuli applied to the nerves of the left and right hind limbs in control tests (**C**) and after the injections (**D**, dynorphin B; **N**, naloxone). The means ± SD of the areas of the initial positive components of the FPs were defined, and the obtained statistical parameters were normalized with respect to the mean value of the control set of records on the given side. The patterns of FP changes varied between experiments and showed differences in the changes on the left and right sides of the spinal cord. Both increases and decreases in the control FPs were observed; the differences were statistically significant in many cases, achieving 0.5–0.75 of the control values.

Two-Way ANOVA was used for the quantitative analysis of the possible injection-dependent changes in the FPs (Table 1). Two factors, the recording side of the spinal cord, left (**L**) or right (**R**); the type of injection, dynorphin B (**D**) or naloxone (**N**), as well as their interaction, were taken into consideration. In most cases, the differences between the sets of parameters under study were statistically significant at the *P* < 0.05 level, although exceptions were also present. The corresponding cells in the Table are highlighted in gray. Using this experimental approach, finding the essential differences between the patterns of FP changes associated with the stimulation of different nerves (GS, FDL or TIB) was not possible. Therefore, based on the similarity of the reactions, all of the tests were treated as a shared population. From a total of 21 tests in 9 experiments, a statistically significant influence of the **Side** factor on the analyzed parameter of the FPs (area of its initial positive component) was not registered in only two cases (the highlighted cells relating to the responses on GS stimulation in experiments 2 and 3; Table 1). In the first case (see also GS reactions in experiments 2 and 3; Figure 2), the dynamics of the FP changes were quite similar on the left and right sides. In the second case, the absence of the side-dependent differences of the reactions might be connected with an increased variability of the FPs on the right side. The independence of the FPs from the **Inj factor** was also observed in two tests (FDL and TIB in experiment 7). A comparison of these results with the general appearance of the bar plots for this experiment (Figure 2) makes it clear that the patterns of the FP changes were quite similar for these nerves. In spite of the asymmetry of the reactions on the left and right sides, the relative change in the FPs was not significant. The weakness of naloxone's influence in the GS tests was associated with a potent side-dependent asymmetry for the dynorphin B injections, whereas the changes in the FPs during FDL and TIB stimulation were essentially different. The interaction of the two factors (**Side** X **Inj**) was not efficient in one of the tests (GS in experiment 2). In this case, there was a high level of similarity in the reactions on different sides of the spinal cord. In spite of the discernible action of the dynorphin B and naloxone injections, both substances diminished the amplitude of the previously recorded reactions with considerable similarity. It could be concluded that both factors (injection type and side of recording), as well as their combination, strongly influenced the amplitude parameters of the recorded FPs.

The Two-Way ANOVA analysis was followed by the *post-hoc* Bonferroni tests presented in Table 2. In this case, it was possible to define the influences of the **Inj** and **Side** factors independently of each other, thus allowing comparison of the statistical significance of changes in a consecutive chain of FP changes (**C–D–N**) independent of each side. Furthermore, the *post-hoc* tests allow the evaluation of the differences between reactions on the left and right sides of the spinal cord. *Post-hoc* testing for the **Side** factor revealed that statistically significant side-dependent differences for the dynorphin B injections were present in 14 of the 21 tests. The differences for the naloxone injections were even more pronounced, and the absence of statistical significance at the *P* < 0.05 level was registered in only one test. Definite conclusions concerning the asymmetrical properties of the reactions on the left and right sides of the spinal cord could be derived from *post-hoc* tests for the **Side** factor. FPs recorded on the left side demonstrated a higher extent of variability in response to both types of injections. The absence of statistically significant changes in the FPs after dynorphin B injections was observed in only one test on the left side, whereas on the right side, these changes were observed in eight tests. For naloxone, these changes were observed in 6 and 10 tests, respectively. Therefore, the injections of dynorphin B and naloxone lead predominantly to noticeable changes in the FPs recorded during stimulation of identical nerves in different hind limbs.

**Table 2:**
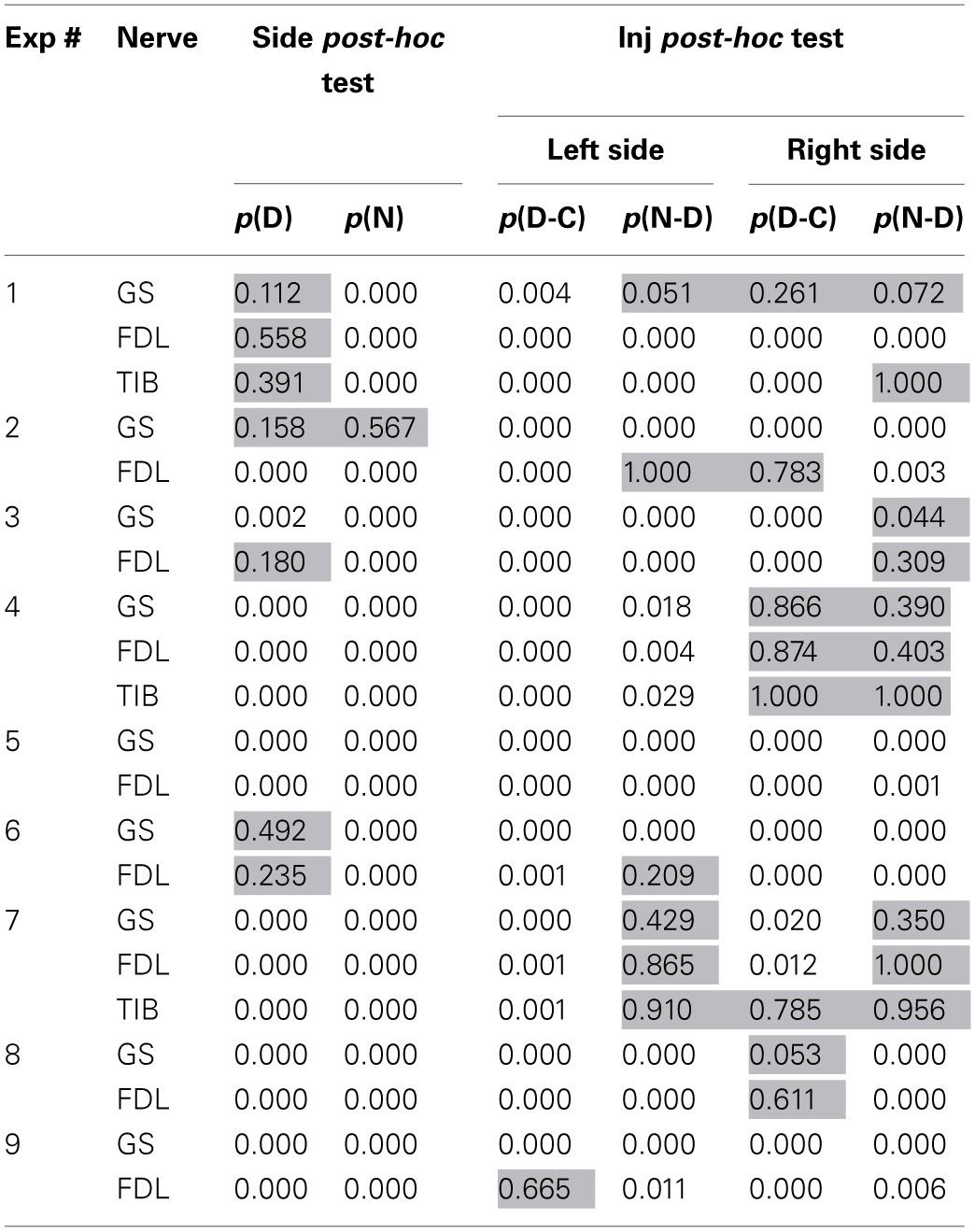
***Post-hoc* analysis of the data for continuation of the ANOVA tests**.

*The post-hoc Bonferroni tests were performed in addition to the Two-Way ANOVA analysis (Table 1) to define the influences of the Inj and Side factors independently of each other; abbreviations: D, dynorphin B, N, naloxone; C, control. Post-hoc tests for Side factor evaluate the probability of side-dependent asymmetry for changes in the FPs with respect to the dynorphin B and naloxone injections; post-hoc tests for ***Inj*** factor define the significance of FP changes in the paired comparisons: D–C and N–D for each side of the spinal cord. The absence of statistical significance at the P < 0.05 level is marked by gray color*.

**Figure 3:**
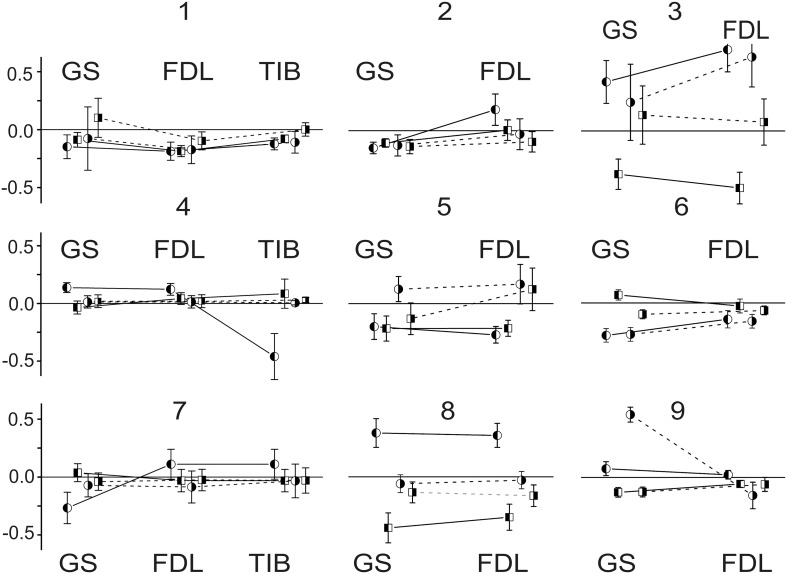
**Comparison of relative changes in statistical parameters in FPs recorded at different sides of spinal cord after dynorphin B and naloxone injections**. The relative changes (m_Dyn_/*m*_Con_ -1) ± (SD_Dyn_ + SD_Con_)/*m*_Con_; (*m*_Nal_ − *m*_Dyn_)/*m*_Con_ ± (SD_Nal_ + SD_Con_)/*m*_Con_ are presented at Y axes of the graphs. The variances from a procedure of subtraction between the two stochastic values are summed. The enumeration of experiments is the same as that used in Figure 2 and in Tables 1, 2. The circles and squares signify the parameters relating to the dynorphin B and naloxone injections, respectively; the left- and right-hand shadowed symbols are related to the records on the left and right sides of spinal cord. Identical parameters are connected by lines in each experiment, the solid and dashed lines correspond to the left and right sides.

A general impression concerning the dynamics of the FP changes for the different nerves in the experiments can be obtained from Figure 3, which shows the relative changes in the statistical parameters of the FPs on the left and right sides of the spinal cord after dynorphin B and naloxone injections. The minor slopes of the connecting lines signify the close similarity of the changes in identical parameters between any pair of nerves in a given experiment. A rise in the slopes indicates an increase in the divergence between the parameters. The experiment presented in Figure 1 (experiment 8 in Figures 2, 3 and Tables 1, 2) can be considered an example of the close conformity observed in the parameters of all the groups. Experiment N4 demonstrates the proximity of the corresponding parameters in the GS and FDL tests and their differences in TIB.

**Figure 4:**
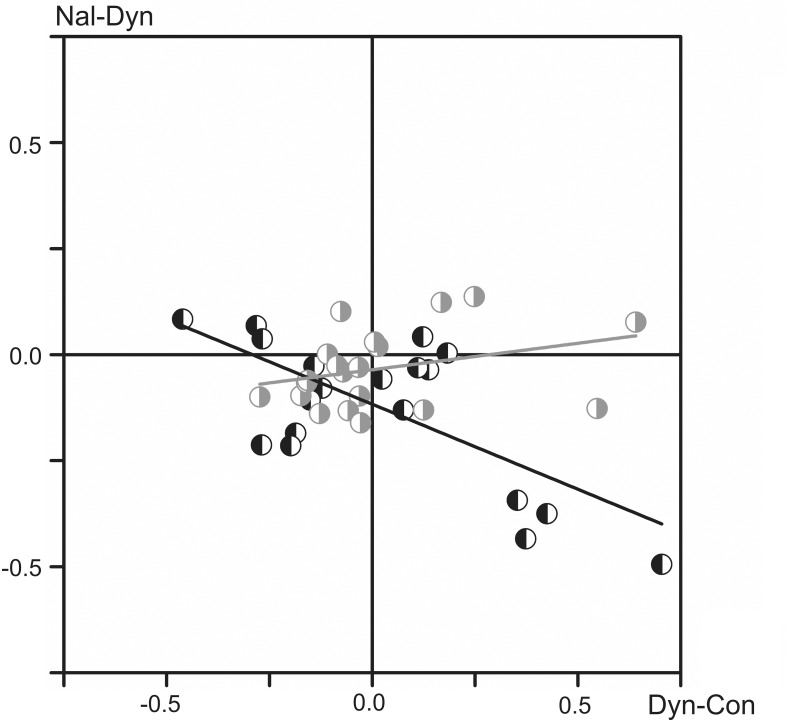
**Analysis of interdependence between the normalized mean values of the FP changes that have been evoked by the injected substances (all tests recorded in nine experiments)**. The data for the left and right sides of the spinal cord are presented separately using the left- or right-hand shadowed symbols in black and gray colors, respectively. The linear regression lines (marked by the corresponding colors) are defined for the two data sets separately, and their parameters are as follows: *r* = −0.68; S (slope) = −0.40 (left side); *r* = 0.31; *S* = 0.12 (right side).

The relationship between the changes in the FPs associated with the two types of injected substances is shown in the scatter plot in Figure 4. The abscissa and ordinate of the plot correspond to the normalized mean values of the FP changes after the dynorphin B and naloxone injections, respectively, and the data for the left and right sides are shown separately, as indicated by different symbols. A statistically significant correlation was recorded only for the population relating to the left side of the spinal cord (ANOVA: *F* value 16.68; Prob > *F*0.0006), while no correlation was registered for the right side (*F* value 2.02; Prob > *F* 0.17). The negative slope of the regression line of the left side of the spinal cord, which extends over the second and fourth quadrants of the plot in Figure 4, may signify a higher probability for FP changes in the opposite direction after the dynorphin B and naloxone injections.

## Discussion

The main inferences of the present study may be associated with the potent effects of dynorphin B and naloxone on the segmental reflex apparatus. We compared FPs recorded bilaterally and symmetrically within deep layers of the spinal cord near the hind limb motoneuronal nuclei using low-resistance glass microelectrodes. In accordance with a general conception of the distribution of potentials in a volume conductor, slow postsynaptic potentials at the membranes of interneurons and motoneurons would indicate a primary source of FPs in spinal cord gray matter, whereas generated spikes must be attenuated (Riddell and Hadian, 2000; Pina et al., 2001). The most notable finding of the present study was the potent modulation of the corresponding segmental reflexes. The patterns of influences exerted by the injected substances were not uniform in the different experiments; both positive and negative changes were observed. The action of the substances was often quite significant, achieving up to a 75% increase and 50% decrease in the analyzed FP parameters in different experiments (Figures 1 through 4). Generally, we did not observe identical responses to stimulation of similar nerves from different hind limbs. Instead, in most cases, a pronounced asymmetry between the reactions on different sides of the spinal cord was observed (Tables 1, 2). To diminish the influence of variability in the recorded potentials, instead of the amplitude parameters of the FPs, the areas of their initial positive components were compared. Performing a normalization procedure on the control values of the FPs allowed comparison of the evoked changes on both sides of the spinal cord.

At present, it is difficult to explain the high degree of variability in the results obtained in different animals. Both the direction and the relative amplitudes of the FP changes after dynorphin B and naloxone injections varied in different animals. Two-Way ANOVA analysis showed that two factors, the injection type and the side of recording, as well as a combination of these two factors, strongly influenced the FPs. *Post-hoc* Bonferroni analysis uncovered statistically significant side-dependent differences in 14 of the 21 tests of the action of dynorphin B and in 20 of the 21 tests for naloxone. However, the directions in these changes, increases or decreases of the FPs, were not associated with a definite side of the spinal cord, left or right. The results indicate that the κ-opioid receptors might be non-uniformly distributed over the neuronal populations transmitting the motor signals from the peripheral afferents of the different hind limbs. Spatial distributions of the receptors on the left and right sides of the spinal cord seem to vary in different animals. Therefore, the existence of lateral asymmetry in these distributions is clear, and a predominance on one sides of the spinal cord in various animals is observed.

The absence of real lateralization (with predominance of action on the left or right side of the spinal cord) in the action of dynorphin B on segmental reflexes in cats could contradict the phenomenon of postural asymmetry induced by endogenous opioid peptides in rats (Chamberlain et al., 1963a,b; Bakalkin, 1989). This discrepancy could be connected to interspecies differences. However, it may also be linked to the different methodologies used in the present study and in the above cited papers to identify the segmental reflexes via corresponding skeletal muscle reactions.

The field potentials recorded in the intermediate zone of spinal gray matter at least partly reflect the activity of the interneurons incorporated in the excitatory and inhibitory pathways to motoneurons, although their involvement seems to be less apparent due to their smaller size compared to motoneurons. In the present study, we observed both increases and decreases of the FPs following dynorphin B and naloxone injections. Due to the strong influence by these substances on κ-opioid receptors, it can be assumed that these receptors are quite non-uniformly distributed over the neuronal populations participating in the transmission of the motor control signals from peripheral afferents to the CNS in cats. Therefore, chemical stimulation of the receptors, or their deactivation by dynorphin B and naloxone injections, could affect different populations of interneurons to produce a combination of excitatory and inhibitory actions on the homonymous motoneurons, which receive predominantly excitation synaptic inflow from stimulated peripheral nerves.

Injections of dynorphin B and naloxone did not produce strictly opposite changes in the FPs; in many cases, such changes were affected identically, i.e., an increase or decrease in the potentials evoked by dynorphin B injection were followed by further change in the same direction after naloxone injection. As follows from Figure 4, which illustrates the interdependence between the changes in the FPs evoked by the two types of injected substances, a statistically significant negative correlation for these changes was registered only on the left side of spinal cord. In the most cases, the Two-Way ANOVA analysis (Table 1) demonstrated statistically significant differences in the dependence of the FP changes on dynorphin B and naloxone injections, as well as the side-dependent asymmetry of these changes. In addition, the patterns of these effects are essentially changed not only in different experiments but also for different pairs of nerves in the same experiments.

Traditionally, the endogenous opioid peptides are considered as evoking predominantly inhibitory effects on neuronal transmission *in vivo* experiments (Simmons and Chavkin, 1996), although recently it has been demonstrated facilitation of spinal reflexes by dynorphin B (Liu et al., 2011). Analysis of action of agonists of κ-opioid receptors in *in vitro* demonstrated both enhancement (Lai et al., 1998), and suppression (Brauneis et al., 1996) of the NMDA receptor-induced depolarization; moreover, mixed actions were also described (Caudle and Dubner, 1998). Action of the antagonists of κ-opioid receptors is also ambiguous, and naloxone can potentiate facilitatory effects evoked by the receptors agonists (Clarke and Ford, 1987). Thus, predominantly antagonistic relationship of dynorphin B and naloxone in their action on κ-opioid receptors (Clarke et al., 1998; Schwarzer, 2009) does not likely signify that these substances should yield strictly opposite actions on the integrated neuronal activity. At least for FPs on the left side of the spinal cord elements, such interdependence was displayed for the normalized mean values of FP changes evoked by dynorphin B and naloxone (Figure 4). Additionally, the absence of the negative correlation between these effects, as in the case of the right side data, may indicate that dynorphin B and naloxone are involved into some additional pathways influencing the neuronal activity in the spinal cord.

## Conclusions

Dynorphin B (an agonist of κ-opioid receptors) and naloxone (an antagonist of opioid receptors) produced significant (up to 75%) changes in the areas of the initial positive components of FPs evoked by sequential stimulation of identical nerves in both hind limbs in spinal zed cats. Two factors, the injection type and the recording side, as well as a combination of these factors, strongly influenced the FP amplitudes. Both the direction of the FP changes and their relative amplitudes were not associated with a definite side of the spinal cord. Therefore, the κ-opioid receptors are distributed inhomogeneously over *the neuronal populations* transmitting the motor signals from peripheral afferents of different hind limbs, thus indicating the presence of effects from the lateral asymmetry that show the predominance of reactions on the left or right sides in various animals.

### Conflict of interest statement

The authors declare that the research was conducted in the absence of any commercial or financial relationships that could be construed as a potential conflict of interest.
